# Supplementary material for: Comparison of Two Efficient Methods for Calculating Partition Functions
Source: Entropy (Basel). 2019 Oct 28;21(11):1050. doi: 10.3390/e21111050 (PMC7514354; doi:10.3390/e21111050)
Supplement: Supplementary file 1 [file entropy-21-01050-s001.pdf]

Supplementary Materials

TABLE S1: The dependence of the pressure (P_{NS}) at different temperatures obtained by the NS and the standard deviations of pressure (SDP_{NS}) of the NS upon the L.

T(K)	L							
	45000		60000		75000		90000	
	P_{NS}(Bar)	SDP_{NS}	P_{NS}(Bar)	SDP_{NS}	P_{NS}(Bar)	SDP_{NS}	P_{NS}(Bar)	SDP_{NS}
25	1128.63	26.01	1104.70	25.98	1106.21	15.71	1108.89	20.92
50	2054.95	53.26	1976.51	23.35	1964.86	22.76	1929.93	25.16
75	2983.09	106.34	2850.54	55.70	2826.54	48.72	2759.18	32.14
100	3927.35	159.91	3735.86	91.87	3692.61	92.81	3575.51	41.55
125	4833.90	209.43	4591.96	114.66	4540.82	114.48	4393.05	53.21
150	5697.33	239.95	5436.13	113.00	5366.85	145.13	5195.22	67.06
175	6505.84	264.96	6235.18	122.19	6168.99	152.24	5978.75	80.61
200	7268.92	247.53	7006.49	112.96	6952.82	167.29	6747.54	93.43
225	8011.09	241.41	7747.88	105.38	7705.80	172.29	7496.92	101.90
250	8726.02	237.59	8475.01	100.01	8412.63	164.78	8220.64	109.64
275	9404.43	214.50	9172.44	99.74	9121.07	163.29	8933.47	117.10
300	10067.28	198.08	9837.85	93.99	9801.02	148.94	9619.66	113.61

TABLE S2: For 500 solid Ar atoms with the density of $1.83 g/cm^3$, the internal energy E_{DIA} and pressure P_{DIA} derived from the DIA, those (E_{NS} and P_{NS}) derived from NS and those (E_{MD} and P_{MD}) obtained from the MD simulations, as well as the relative difference of internal energy ($RDE_{DIA} = \left|\frac{E_{DIA}-E_{MD}}{E_{MD}}\right|$, $RDE_{NS} = \left|\frac{E_{NS}-E_{MD}}{E_{MD}}\right|$) and pressure ($RDP_{DIA} = \left|\frac{P_{DIA}-P_{MD}}{P_{MD}}\right|$, $RDP_{NS} = \left|\frac{P_{NS}-P_{MD}}{P_{MD}}\right|$), together with the relative standard deviations of internal energy (RSDE) and pressure (RSDP) of the MD simulations.

T(K)	Internal Energy (eV)					
	E_{DIA}	E_{NS}	E_{MD}	RDE_{DIA}(%)	RDE_{NS}(%)	RSDE(%)
25	-40.24	-39.76	-40.61	0.90	2.08	0.06
50	-37.07	-36.48	-37.49	1.11	2.69	0.11
75	-33.93	-33.14	-34.41	1.39	3.68	0.20
100	-30.81	-29.76	-31.37	1.78	5.13	0.28
125	-27.72	-26.44	-28.35	2.24	6.74	0.40
150	-24.64	-23.18	-25.35	2.79	8.56	0.58
175	-21.58	-19.92	-22.34	3.40	10.85	0.78
200	-18.54	-16.76	-19.35	4.19	13.42	1.05
225	-15.52	-13.66	-16.33	4.97	16.37	1.56
250	-12.51	-10.58	-13.31	5.99	20.45	2.08
275	-9.51	-7.57	-10.31	7.72	26.52	2.93
300	-6.53	-4.63	-7.15	8.63	35.18	5.30
Average Relative Difference	-	-	-	3.32	10.59	-

T(K)	Pressure (Bar)					
	P_{DIA}	P_{NS}	P_{MD}	RDP_{DIA}(%)	RDP_{NS}(%)	RSDP(%)
25	921.02	1121.58	898.29	2.53	24.86	1.78
50	1734.98	1976.51	1684.51	3.00	17.33	1.81
75	2526.03	2850.54	2445.52	3.29	16.56	2.02
100	3304.03	3735.86	3182.92	3.80	17.37	1.98
125	4069.89	4591.96	3909.10	4.11	17.47	2.05
150	4823.69	5436.13	4623.47	4.33	17.58	2.12
175	5566.21	6235.18	5333.56	4.36	16.90	2.23
200	6298.59	7006.49	6035.03	4.37	16.10	2.24
225	7021.98	7747.88	6749.18	4.04	14.80	2.39
250	7737.37	8475.01	7460.21	3.72	13.60	2.30
275	8445.58	9172.44	8158.80	3.51	12.42	2.27
300	9147.27	9837.85	8925.57	2.48	10.22	2.45
Average Relative Difference	-	-	-	3.63	16.27	-

TABLE S3: The system of 500 solid Ar atoms with the density $2.13 g/cm^3$

T(K)	Internal Energy (eV)					
	E_{DIA}	E_{NS}	E_{MD}	RDE_{DIA}(%)	RDE_{NS}(%)	RSDE(%)
25	-34.53	-33.64	-34.85	0.90	3.46	0.04
50	-31.32	-30.30	-31.67	1.10	4.34	0.09
75	-28.13	-26.96	-28.53	1.39	5.50	0.17
100	-24.95	-23.60	-25.40	1.78	7.11	0.25
125	-21.78	-20.23	-22.29	2.28	9.25	0.39
150	-18.63	-16.84	-19.21	3.00	12.34	0.53
175	-15.49	-13.47	-16.13	4.01	16.54	0.73
200	-12.35	-10.09	-13.09	5.59	22.90	1.02
225	-9.23	-6.72	-10.03	7.90	32.93	1.54
250	-6.12	-3.40	-6.98	12.22	51.30	2.53
275	-3.02	-0.06	-3.95	23.46	98.55	5.05
300	0.07	3.27	-0.92	107.59	455.18	23.28
Average Relative Difference	-	-	-	4.02	16.57	-

T(K)	Pressure (Bar)					
	P_{DIA}	P_{NS}	P_{MD}	RDP_{DIA}(%)	RDP_{NS}(%)	RSDP(%)
25	9843.23	10296.19	9911.79	0.69	3.88	0.15
50	10746.49	11221.55	10790.09	0.40	4.00	0.27
75	11640.10	12174.96	11653.44	0.11	4.48	0.42
100	12523.75	13136.00	12502.89	0.17	5.06	0.51
125	13398.33	14097.03	13344.49	0.40	5.64	0.62
150	14264.77	15069.83	14173.19	0.65	6.33	0.73
175	15123.68	16029.05	14992.18	0.88	6.92	0.76
200	15975.52	16989.84	15798.84	1.12	7.54	0.82
225	16820.67	17942.32	16609.66	1.27	8.02	0.89
250	17659.52	18875.31	17413.37	1.41	8.40	0.93
275	18492.41	19813.46	18205.75	1.57	8.83	1.01
300	19319.69	20741.79	18997.73	1.69	9.18	1.04
Average Relative Difference	-	-	-	0.86	6.52	-

TABLE S4: The system of 500 solid Ar atoms with the density $2.43 g/cm^3$

T(K)	Internal Energy (eV)					
	E_{DIA}	E_{NS}	E_{MD}	$RDE_{DIA}(\%)$	$RDE_{NS}(\%)$	$RSDE(\%)$
25	-14.85	-13.35	-15.01	1.07	11.05	0.07
50	-11.63	-10.00	-11.82	1.58	15.38	0.20
75	-8.42	-6.67	-8.64	2.59	22.79	0.39
100	-5.21	-3.34	-5.48	4.91	39.00	0.85
125	-2.01	-0.01	-2.33	13.73	99.37	2.57
150	1.18	3.32	0.80	47.17	315.09	9.10
175	4.36	6.66	3.93	11.07	69.53	2.20
200	7.54	10.02	7.04	7.10	42.26	1.50
225	10.71	13.35	10.15	5.53	31.52	1.12
250	13.88	16.70	13.25	4.76	26.05	0.98
275	17.04	20.08	16.34	4.29	22.91	0.85
300	20.19	23.49	19.42	3.99	21.01	0.78
Average Relative Difference	-	-	-	5.51	36.44	-

T(K)	Pressure (Bar)					
	P_{DIA}	P_{NS}	P_{MD}	$RDP_{DIA}(\%)$	$RDP_{NS}(\%)$	$RSDP(\%)$
25	28586.96	29387.89	28835.02	0.86	1.92	0.05
50	29574.68	30431.79	29805.56	0.77	2.10	0.11
75	30554.78	31469.33	30766.73	0.69	2.28	0.16
100	31525.12	32506.44	31718.99	0.61	2.48	0.21
125	32486.14	33542.00	32662.35	0.54	2.69	0.25
150	33439.06	34581.77	33599.56	0.48	2.92	0.31
175	34384.91	35619.67	34530.86	0.42	3.15	0.35
200	35324.46	36666.90	35455.98	0.37	3.42	0.40
225	36258.34	37701.12	36375.64	0.32	3.64	0.44
250	37187.13	38740.79	37287	0.27	3.90	0.48
275	38111.38	39796.98	38194.52	0.22	4.20	0.49
300	39031.58	40870.72	39096.9	0.17	4.54	0.55
Average Relative Difference	-	-	-	0.48	3.10	-

TABLE S5: The system of 500 solid Ar atoms with the density $2.98 g/cm^3$

T(K)	Internal Energy (eV)			
	E_{DIA}	E_{MD}	$RDE_{DIA}(\%)$	$RSDE(\%)$
25	79.15	79.66	0.64	0.64
50	82.38	82.88	0.61	1.19
75	85.60	86.09	0.57	1.89
100	88.82	89.29	0.53	2.42
125	92.04	92.48	0.48	2.97
150	95.25	95.67	0.44	3.61
175	98.46	98.86	0.40	4.05
200	101.67	102.03	0.36	4.56
225	104.87	105.20	0.31	4.96
250	108.08	108.37	0.27	5.31
275	111.28	111.53	0.22	5.80
300	114.47	114.68	0.18	5.97
Average Relative Difference	-	-	0.48	-

T(K)	Pressure (Bar)			
	P_{DIA}	P_{MD}	$RDP_{DIA}(\%)$	$RSDP(\times 10^{-4}\%)$
25	111497.83	112504.41	0.89	0.06
50	112660.49	113658.22	0.88	0.12
75	113821.29	114807.60	0.86	0.19
100	114980.14	115953.13	0.84	0.24
125	116137.06	117094.17	0.82	0.32
150	117292.05	118231.81	0.79	0.38
175	118445.07	119362.42	0.77	0.43
200	119596.09	120493.18	0.74	0.50
225	120745.02	121617.46	0.72	0.56
250	121891.76	122738.38	0.69	0.61
275	123036.22	123857.38	0.66	0.67
300	124178.33	124971.82	0.63	0.75
Average Relative Difference	-	-	0.78	-

TABLE S6: The system of 4000 solid Ar atoms with the density $1.83 g/cm^3$

T(K)	Internal Energy (eV)			
	E_{DIA}	E_{MD}	$RDE_{DIA}(\%)$	$RSDE(\%)$
25	-321.93	-324.82	0.89	0.02
50	-296.56	-299.78	1.07	0.04
75	-271.43	-275.13	1.35	0.07
100	-246.49	-250.74	1.69	0.10
125	-221.73	-226.57	2.14	0.15
150	-197.12	-202.49	2.65	0.20
175	-172.67	-178.45	3.24	0.27
200	-148.34	-154.48	3.97	0.39
225	-124.15	-130.42	4.81	0.51
250	-100.07	-106.07	5.65	0.79
275	-76.10	-81.31	6.40	1.22
300	-52.24	-54.47	4.11	2.23
Average Relative Difference	-	-	3.16	

T(K)	Pressure (Bar)			
	P_{DIA}	P_{MD}	$RDP_{DIA}(\%)$	$RSDP(\%)$
25	922.15	899.56	2.51	0.63
50	1737.22	1688.25	2.90	0.66
75	2529.36	2449.22	3.27	0.65
100	3308.44	3190.91	3.68	0.72
125	4075.35	3916.07	4.07	0.73
150	4830.19	4632.22	4.27	0.76
175	5573.74	5342.97	4.32	0.76
200	6307.14	6046.42	4.31	0.80
225	7031.52	6752.28	4.14	0.78
250	7747.90	7472.19	3.69	0.85
275	8457.09	8211.81	2.99	0.88
300	9159.76	9064.18	1.05	0.91
Average Relative Difference	-	-	3.43	

TABLE S7: The system of 4000 solid Ar atoms with the density $2.13 g/cm^3$

T(K)	Internal Energy (eV)			
	E_{DIA}	E_{MD}	$RDE_{DIA}(\%)$	$RSDE(\%)$
25	-276.25	-278.74	0.89	0.02
50	-250.60	-253.30	1.07	0.04
75	-225.06	-228.08	1.33	0.06
100	-199.62	-203.03	1.68	0.09
125	-174.28	-178.14	2.17	0.13
150	-149.03	-153.41	2.85	0.18
175	-123.89	-128.78	3.80	0.28
200	-98.83	-104.25	5.19	0.38
225	-73.87	-79.78	7.41	0.58
250	-48.98	-55.41	11.60	0.89
275	-24.18	-31.14	22.36	1.80
300	0.56	-6.83	108.16	9.26
Average Relative Difference	-	-	5.49	

T(K)	Pressure (Bar)			
	P_{DIA}	P_{MD}	$RDP_{DIA}(\%)$	$RSDP(\%)$
25	9855.36	9913.45	0.59	0.06
50	10759.82	10793.84	0.32	0.11
75	11654.63	11658.68	0.03	0.15
100	12539.48	12510.81	0.23	0.18
125	13415.25	13351.81	0.48	0.21
150	14282.86	14181.26	0.72	0.24
175	15142.94	15001.75	0.94	0.27
200	15995.95	15815.99	1.14	0.29
225	16842.26	16623.92	1.31	0.33
250	17682.26	17425.94	1.47	0.32
275	18516.29	18220.64	1.62	0.36
300	19344.70	19015.42	1.73	0.37
Average Relative Difference	-	-	0.88	

TABLE S8: The system of 4000 solid Ar atoms with the density $2.43 g/cm^3$

T(K)	Internal Energy (eV)			
	E_{DIA}	E_{MD}	$RDE_{DIA}(\%)$	$RSDE(\%)$
25	-118.78	-120.02	1.03	0.04
50	-93.04	-94.44	1.49	0.08
75	-67.34	-69.01	2.41	0.15
100	-41.70	-43.68	4.53	0.34
125	-16.11	-18.46	12.74	0.98
150	9.43	6.67	41.30	3.23
175	34.91	31.72	10.07	0.81
200	60.34	56.69	6.43	0.51
225	85.71	81.58	5.07	0.41
250	111.03	106.41	4.35	0.33
275	136.30	131.18	3.91	0.31
300	161.52	155.90	3.60	0.29
Average Relative Difference	-	-	8.08	

T(K)	Pressure (Bar)			
	P_{DIA}	P_{MD}	$RDP_{DIA}(\times 10^{-2}\%)$	$RSDP(\%)$
25	28589.29	28836.72	0.81	0.02
50	29577.01	29809.12	0.79	0.04
75	30557.12	30771.36	0.76	0.06
100	31527.46	31725.51	0.74	0.08
125	32488.48	32671.63	0.72	0.10
150	33441.40	33610.59	0.70	0.11
175	34387.26	34542.62	0.68	0.13
200	35326.82	35468.73	0.67	0.14
225	36260.70	36388.17	0.65	0.16
250	37189.49	37303.43	0.64	0.16
275	38113.74	38213.27	0.62	0.18
300	39033.96	39119.07	0.61	0.19
Average Relative Difference	-	-	0.70	

TABLE S9: The system of 4000 solid Ar atoms with the density $2.98 g/cm^3$

T(K)	Internal Energy (eV)			
	E_{DIA}	E_{MD}	$RDE_{DIA}(\%)$	$RSDE(\times 10^{-2}\%)$
25	632.53	637.36	0.76	0.43
50	658.34	663.13	0.72	0.71
75	684.12	688.83	0.68	0.99
100	709.88	714.49	0.65	1.27
125	735.61	740.10	0.61	1.52
150	761.33	765.65	0.56	1.79
175	787.02	791.15	0.52	1.92
200	812.68	816.62	0.48	2.13
225	838.33	842.03	0.44	2.38
250	863.95	867.41	0.40	2.60
275	889.55	892.74	0.36	2.87
300	915.14	918.04	0.32	3.11
Average Relative Difference	-	-	0.54	

T(K)	Pressure (Bar)			
	P_{DIA}	P_{MD}	$RDP_{DIA}(\%)$	$RSDP(\times 10^{-2}\%)$
25	111619.41	112506.48	0.92	0.67
50	112783.61	113662.36	0.91	1.24
75	113945.95	114813.95	0.89	1.86
100	115106.37	115960.93	0.87	2.49
125	116264.86	117103.93	0.85	3.01
150	117421.40	118242.72	0.83	3.63
175	118575.99	119377.37	0.81	4.13
200	119728.57	120508.96	0.78	4.80
225	120879.06	121636.21	0.76	5.34
250	122027.35	122760.13	0.73	5.84
275	123173.37	123880.66	0.71	6.39
300	124317.04	124998.01	0.68	7.15
Average Relative Difference	-	-	0.68	
